# Metabolic-stress-induced mitochondrial calcium dysregulation: a central hub in diabetic cardiomyopathy pathogenesis and treatment

**DOI:** 10.3389/fendo.2025.1696344

**Published:** 2025-12-04

**Authors:** Siqi Deng, Fatemeh Tayefi, Yunpeng Jin

**Affiliations:** Department of Cardiology, The Fourth Affiliated Hospital of School of Medicine, and International School of Medicine, International Institutes of Medicine, Zhejiang University, Yiwu, Zhejiang, China

**Keywords:** diabetic cardiomyopathy, mitochondrial calcium, calcium homeostasis, mitochondrial dysfunction, heart failure, therapeutic targets

## Abstract

Diabetic cardiomyopathy (DCM), as a devastating complication of diabetes mellitus (DM), arises from a complex interplay between systemic metabolic derangements and myocardial vulnerability. While hyperglycemia, lipotoxicity, and insulin resistance are established drivers of cardiac dysfunction, the precise mechanisms linking these metabolic insults to cardiac dysfunction remain elusive. Recent evidence suggests that the dysregulation of mitochondrial calcium homeostasis plays a critical role in integrating diabetic metabolic stress and cardiomyocyte fate. This review synthesizes recent advances in understanding how mitochondrial calcium mishandling—encompassing impaired uptake, excessive release, and buffering failure—orchestrates the pathological triad of bioenergetic deficit, oxidative stress, and cell death in DCM. We delve into the molecular mechanisms underpinning this dysregulation, highlighting its interplay with the diabetic metabolic milieu. Furthermore, we critically evaluate novel therapeutic strategies targeting mitochondrial calcium fluxes, including the inhibition of the mitochondrial calcium uniporter (MCU), the activation of the mitochondrial Na^+^/Ca^2+^/Li^+^ exchanger (NCLX), and the modulation of the mitochondrial permeability transition pore (mPTP), discussing their clinical translation potential and existing challenges. By reframing DCM through the lens of mitochondrial calcium homeostasis, this review not only synthesizes current knowledge but also provides a critical comparison of emerging therapeutic strategies and evaluates the formidable challenges in their clinical translation, thereby bridging the gap between endocrine metabolism and cardiac pathophysiology and offering nuanced perspectives for biomarker discovery and stage-specific interventions.

## Highlights

- Mitochondrial calcium (Ca^2+^) imbalance is a core mechanism linking systemic metabolic stress to cardiac dysfunction in diabetes.

- Diabetic conditions (hyperglycemia, advanced glycation end products (AGEs), and oxidative stress) directly impair the molecular machinery of mitochondrial calcium uptake (MCU) and release (mPTP, NCLX).

- Mitochondrial calcium imbalance triggers a vicious cycle of energy crisis, oxidative damage, and cardiomyocyte death, culminating in heart failure.

- Therapeutic strategies targeting mitochondrial calcium fluxes (e.g., cardiac-specific MCU inhibitors, NCLX activators) show promise in preclinical models.

- Translating these strategies to the clinic requires stage-specific approaches and reliable biomarkers of mitochondrial function.

## 1 Introduction

### 1.1 Background

Diabetic cardiomyopathy (DCM), a pathophysiological condition induced by diabetes mellitus (DM), is a significant contributor to heart failure (HF) (1). Leading global health organizations, including the World Health Organization (WHO), the American Heart Association (AHA), and the International Diabetes Federation (IDF), have identified DCM as a predominant cause of mortality in patients with diabetes (2–4).

Recent population-based studies report that 12–17% of patients with type 2 diabetes (T2D) are diagnosed with DCM, while systematic cardiac phenotyping reveals subclinical cardiac dysfunction in over 50% of these patients (5, 6). Prospective cohort data indicate that 29% of asymptomatic T2D patients progress to overt DCM within 5 years, primarily driven by metabolic stress and microvascular injury (7). The trajectory involves progressive myocardial fibrosis and inflammation, ultimately culminating in HF (8). Notably, DCM is associated with a 75% higher risk of cardiovascular mortality than non-diabetic HF, after adjustment for comorbidities (9). The persistent and significant contribution of diabetic cardiomyopathy to the global burden of cardiovascular mortality has been further emphasized in recent large-scale epidemiological studies (10).

The global burden of DCM has mirrored the diabetes pandemic, with age-standardized prevalence rising by 7.9% annually between 2017 and 2022. This increase strongly correlates with regional T2D growth rates (ρ=0.91, p<0.001) (11). More recent estimates from the Global Burden of Disease Study underscore the escalating burden, highlighting the urgent need for targeted interventions (12). Mechanistic drivers (e.g., mitochondrial dysfunction, lipotoxicity) remain therapeutic targets; however, no disease-modifying therapies currently exist (13, 14). Ongoing research continues to delineate the intricate mechanisms, including mitochondrial calcium dysregulation, that underpin the progression from diabetes to heart failure (15). This therapeutic gap is further emphasized in contemporary reviews, which call for a deeper understanding of the fundamental pathophysiological processes, such as mitochondrial quality control and ion homeostasis (16).

Mitochondrial calcium (Ca^2+^) imbalance is a key pathophysiological link between diabetic metabolic derangements and myocardial dysfunction. Given the multitude of Ca^2+^-handling proteins that regulate the Ca^2+^ transients in cardiomyocytes, DCM has been shown to disrupt these proteins through altered expression, function, or both (17). Mitochondria buffer cytosolic Ca^2+^ concentrations, thereby playing a central role in cardiac excitation-contraction coupling (ECC) (18, 19). Impairment of this mitochondrial function compromises cardiac contractility and contributes to cardiac dysfunction (18). In diabetic mice, studies have demonstrated impaired cardiac mitochondrial function associated with ultrastructural defects (20). Conversely, under pathological conditions—such as ischemia, infarction, and pressure overload—cellular stress induces excessive cytosolic Ca^2+^ accumulation, leading to mitochondrial Ca^2+^ overload. This overload increases reactive oxygen species (ROS) generation, disrupts mitochondrial membrane potential (ΔΨ), and promotes opening of the mitochondrial permeability transition pore (mPTP), accompanied by ATP depletion (21, 22). Collectively, these events trigger cardiomyocyte death and progressive cardiac dysfunction. Therefore, maintaining the mitochondrial Ca^2+^ homeostasis is essential for cardiomyocyte survival and functional integrity. Reflecting this, mitochondrial calcium homeostasis is increasingly recognized as a central integrator of metabolic stress and cardiac dysfunction in diabetes, bridging systemic derangements to myocardial injury (23).

This review summarizes recent advances in mitochondrial Ca^2+^homeostasis, emphasizing its critical role in cardiac Ca^2+^ regulation. It also outlines emerging therapeutic strategies targeting mitochondrial Ca^2+^ pathways for DCM treatment and prevention.

## 2 Mitochondrial calcium homeostasis: basics and regulatory mechanisms

### 2.1 Mechanisms of mitochondrial calcium uptake and release

In general, the physiological concentration of mitochondrial Ca^2+^ is tightly regulated by two major operative regulatory systems that either release Ca^2+^ into the cytosol or take up Ca^2+^ from the cytosol. Voltage-dependent anion channel 1 (VDAC1), located on the outer mitochondrial membrane, acts as the first gatekeeper of Ca^2+^ entering mitochondria (24). Initially, Ca^2+^ accumulates in the mitochondrial intermembrane space (IMS), and then, it enters the mitochondrial matrix through the mitochondrial Ca^2+^ uniporter (MCU). By interacting with various regulatory proteins, such as calcium uptake proteins (MICUs), the MCU forms a functional complex that precisely controls the uptake of Ca^2+^ into the matrix. Conversely, Ca^2+^ stored in the matrix can be transported back to IMS through Na^+^/Ca^2+^ (NCX) or H^+^/Ca^2+^exchangers (25), as well as by mPTP opening when the cell is under stress (26, 27). Although Ca^2+^ uptake and release appear to be two independent processes, some studies indicate that an interlink may exist between them. For example, mitochondrial Ca^2+^ uptake may affect the initiation and activation of the releasing system, particularly mPTP opening during stress (28–30). In addition to these conventional regulatory apparatuses, newly identified proteins are being discovered that contribute to mitochondrial Ca^2+^ homeostasis and cardiac mitochondrial function. One such example is valosin-containing protein (VCP), an AAA+ ATPase that has been recently implicated in the regulation of both mitochondrial calcium uptake (by modulating MCU complex stability) and mPTP opening under stress conditions (31).

#### 2.1.1 Mitochondrial calcium uptake

Mitochondria possess a low-affinity, high-capacity calcium ion uptake system, primarily mediated by the MCU (32). MCU is present in almost all cell types and functions as a highly selective calcium channel responsible for Ca^2+^ uptake in mitochondria (33). There are two transmembrane domains located in the inner mitochondrial membrane (IMM), with both the N-terminal and C-terminal domains facing the mitochondrial matrix. The N-terminal domain (NTD) is believed to regulate Ca^2+^ uptake rate (34). Although studies on the molecular nature of the MCU complex began in the 1970s, MCU itself was not identified and confirmed as an essential component of the MCU until 2011 (35–37). Since then, the structure of the MCU has been elucidated in various species, including fungi, C. elegans, zebrafish, and humans (33). The transmembrane and NTD structures are conserved across species, highlighting their essential role in mitochondrial Ca^2+^ homeostasis. Over the years of effort, the core components of the MCU complex (MCU, EMRE, MICU1, MICU2) and associated regulatory factors (such as MCUR1 and MICU3) have been identified. Cryo-electron microscopy technology has specifically elucidated the structure of the MCU-EMRE core channel, offering a molecular foundation for comprehending its functional mechanisms. The identification of MICU1 and MICU2 as gatekeepers that prevent Ca^2+^ uptake under resting conditions represented a breakthrough in understanding the regulation of the uniporter (38). Wang, Y., and his team further elucidated the molecular basis of the MICU1/2 gating role using cryo-electron microscopy (39).

##### 2.1.1.1 Cytosolic low-calcium state ([Ca^2+^]₋ < 1–3 μM)

Biological systems feature exquisitely designed molecular switches. The response of MICU1/2 to calcium ions is not a binary “on/off” switch but rather the result of graded conformational changes. At calcium concentrations below 1 μM, MICU1/2 stably blocks the channel; between 1 and 3 μM, it partially opens; and at approximately 3 μM, the channel becomes fully open (enabling efficient calcium uptake). Thus, activation occurs over a concentration range rather than at a single threshold. The MICU1/MICU2 heterodimer (or, in some cases, MICU1 homodimers) interacts via its positively charged domain with negatively charged regions near the entrance of the MCU-EMRE pore. This physical blocking effect significantly reduces the MCU channel’s affinity for Ca^2+^ (manifested as an increased Kd), effectively closing the channel and preventing leakage of low-level Ca^2+^ into the mitochondria under resting conditions. This regulation is crucial for maintaining matrix ion homeostasis and energy efficiency.

##### 2.1.1.2 Cytosolic elevated-calcium state ([Ca^2+^]₋ > 10 μM)

When cytosolic (or intermembrane space) [Ca^2+^] levels rise, Ca^2+^ binds to the EF-hand domains of MICU1 and MICU2. Ca^2+^ binding induces a significant conformational change in MICU1, weakening or abolishing the physical blockade of the MCU pore by the MICU1/MICU2 complex. As a result, the channel “gate” opens, significantly enhancing the MCU channel’s affinity for Ca^2+^. This allows efficient and rapid Ca^2+^ uptake into the mitochondrial matrix, supporting cellular processes such as energy production and signal transduction.

#### 2.1.2 Mitochondrial calcium release

The mechanisms of mitochondrial calcium release are primarily categorized into the following three types:

##### 2.1.2.1 mPTP

The mPTP is a non-selective, high-conductance channel in the inner mitochondrial membrane. Its sustained opening is a catastrophic event, fundamentally altering mitochondrial homeostasis. While it can lead to the release of Ca^2+^ and other small molecules, its primary pathological role is to cause the irreversible collapse of the ΔΨm, uncoupling oxidative phosphorylation and leading to ATP depletion. Furthermore, osmotic imbalance causes mitochondrial swelling and rupture of the outer membrane, releasing pro-apoptotic factors like cytochrome c, thereby initiating cell death pathways (26, 27, 40). Transient opening leads to the release of calcium ions and small molecules. The molecular composition of mPTP has been a subject of significant recent controversy. The classical model previously described the mPTP as comprising three components: adenine nucleotide translocase (ANT—located in the inner membrane), the voltage-dependent anion channel (VDAC—located in the outer membrane), and cyclophilin D (CypD—a matrix protein, regulatory subunit). CypD binding to ANT promotes pore opening (41). However, recent studies propose a revised model: compelling recent evidence strongly points towards the F_0_F_1_-ATP synthase—specifically its dimers, oligomers, or the c-subunit ring—possibly forming the core pore channel. Genetic knockout experiments have shown that the absence of ANT or VDAC does not completely prevent mPTP opening (42), whereas experimental targeting of F-ATP synthase effectively modulates the mPTP (43).

##### 2.1.2.2 Mitochondrial Na^+^/Ca^2+^ exchanger

Under specific conditions (e.g., decreased cytosolic Ca^2+^ levels, reduced ΔΨm), mitochondria can also release calcium via the sodium-calcium exchanger (38). This exchanger represents the primary “physiological” pathway for mitochondrial calcium efflux. It utilizes the Na^+^ electrochemical gradient across the inner membrane (positive outside, negative inside, and high cytosolic Na^+^ concentration) to actively exchange matrix Ca^2+^ for cytosolic Na^+^ at a stoichiometry of 3 Na^+^: 1 Ca^2+^. The primary physiological pathway for mitochondrial Ca^2+^ efflux is mediated by the mitochondrial Na^+^/Ca^2+^ exchanger (mNCX), whose molecular identity is the Na^+^/Ca^2+^/Li^+^ exchanger (NCLX), encoded by the SLC8B1 gene (44).

##### 2.1.2.3 Rapid mode release

In response to brief, high-frequency cytosolic Ca^2+^ pulses, mitochondria exhibit an extremely rapid calcium release (and uptake), far exceeding the rate mediated by mNCX. This release is independent of the mPTP, and while its molecular identity remains an area of active investigation, it represents a distinct mode of mitochondrial Ca²^+^ flux (22).

### 2.2 Mitochondrial Ca^2+^ buffering

Mitochondrial Ca^2+^ buffering mechanisms (such as calcium phosphate precipitation) are key processes for maintaining matrix calcium homeostasis. They temporarily sequester excess Ca^2+^ in the form of chemical precipitation, thereby preventing cellular damage caused by calcium overload.

Regulatory factors, such as the phosphate transporter (PiC), are part of this process. Kwong, J. Q., and colleagues, using a cardiac-specific model of PiC knockout mice (cPiC-KO), demonstrated the following pathway:

PiC deficiency → Reduced mitochondrial phosphate (Pi) uptake → Inhibition of calcium phosphate precipitation formation → Mitigation of respiratory chain damage caused by calcium overload → →Maintenance of ATP synthesis.

Furthermore, cPiC-KO mice exhibited a 60% reduction in myocardial infarct size and improved cardiac function (LVEF improved by 35%) following ischemia-reperfusion (I/R) injury (45).

It has been reported that the mitochondrial calcium buffering mechanism can prevent mPTP opening triggered by calcium oscillations, such as those occurring during systole in cardiomyocytes (46).

### 2.3 Core functions of mitochondrial calcium signaling

Mitochondrial Ca^2+^ signaling orchestrates cellular homeostasis through three cardinal mechanisms: optimization of bioenergetic output via TCA cycle and oxidative phosphorylation (OXPHOS) regulation, bidirectional modulation of ROS homeostasis, and regulation of cell death pathways via the mPTP. Precise spatiotemporal control of this signaling axis is indispensable for physiological function; its dysregulation precipitates metabolic insufficiency, oxidative injury, or apoptotic cell death.

#### 2.3.1 Regulation of energy metabolism

Calcium influx into the mitochondrial matrix serves as a critical activator of rate-limiting dehydrogenases within the tricarboxylic acid (TCA) cycle. Specifically, Ca^2+^ directly stimulates:

Pyruvate dehydrogenase complex (PDH), relieving inhibitory constraints to accelerate pyruvate decarboxylation to acetyl-CoA (47); isocitrate dehydrogenase (IDH3) (48); and α-ketoglutarate dehydrogenase complex (OGDH) (49).

This concerted activation markedly elevates NADH and FADH_2_ production. These electron carriers subsequently fuel the electron transport chain (ETC), augmenting the proton gradient (Δψm) across the inner mitochondrial membrane. The resulting hyperpolarization potentiates ATP synthase activity, substantially enhancing OXPHOS efficiency (47). Critically, mitochondrial Ca^2+^ uptake through the MCU provides a rapid transduction mechanism that synchronizes mitochondrial energy production with cytosolic Ca^2+^ transients evoked by physiological stimuli (e.g., hormonal signaling or excitation-contraction coupling). This enables real-time matching of mitochondrial ATP production with cellular energy demand (49).

#### 2.3.2 Bidirectional control of ROS

Mitochondrial Ca^2+^ exerts context-dependent effects on redox balance. At physiological concentrations, Ca^2+^ attenuates ROS generation through dual mechanisms: (i) enhanced ATP synthesis mitigates ETC over-reduction, limiting electron leakage; and (ii) activation of the NRF2 antioxidant pathway upregulates mitochondrial ROS-scavenging systems, including manganese superoxide dismutase (MnSOD) and peroxiredoxin 3 (Prx3) (50). Conversely, pathological Ca^2+^ overload induces oxidative stress via (i) structural impairment of ETC complexes (notably Complex I), exacerbating electron escape; (ii) mPTP induction culminating in Δψm dissipation and catastrophic ROS release (51); (iii) context-dependent activation of pro-oxidant enzymes (e.g., glycerol-3-phosphate dehydrogenase) (52); and (iv) suppression of endogenous antioxidant capacity (53).

#### 2.3.3 Apoptotic regulation via mPTP

Matrix Ca^2+^ accumulation serves as an essential trigger for mPTP opening, with co-factors (elevated ROS, diminished Δψm) synergistically lowering its activation threshold. Pore induction initiates a lethal cascade: mitochondrial swelling and irreversible Δψm collapse terminate OXPHOS, while efflux of pro-apoptotic factors (cytochrome *c*, apoptosis-inducing factor) activates caspase-dependent apoptosis (40). Key regulatory components include CypD, whose Ca^2+^-facilitated binding to the ATP synthase c-ring promotes pore formation (54), and modulatory proteins adenine nucleotide translocator (ANT) and phosphate carrier (PiC) that fine-tune mPTP sensitivity (55). The susceptibility to mPTP-mediated cell death is profoundly influenced by the systemic metabolic milieu. AMPK is a crucial integrator of energy and oxidative stress, critically regulates cardiac tolerance to ischemia/reperfusion injury, and its dysregulation in diabetes exacerbates mPTP-dependent cell death (56).

## 3 Mitochondrial calcium homeostasis dysregulation in DCM

### 3.1 Mitochondrial Ca^2+^ dysregulation phenomena observed in both preclinical and clinical studies of DCM include specific manifestations and consequences resulting from mitochondrial Ca^2+^ overload or impaired uptake

#### 3.1.1 Core dysregulation phenomena (preclinical and clinical evidence)

Reduced Mitochondrial Ca^2+^ Uptake Studies have demonstrated the following: In preclinical models of DCM, such as models (db/db mice, STZ rats), exhibit decreased baseline mitochondrial Ca^2+^ content in cardiomyocytes and significantly attenuated mitochondrial Ca^2+^ elevation following β-adrenergic stimulation (17, 57).

Conversely, evidence from human DCM comes from myocardial biopsies of T2DM patients, which reveal reduced MCU protein expression and impaired mitochondrial Ca^2+^ buffering capacity (58). This phenomenon is attributed to direct hyperglycemic impairment: high glucose reduces the myocardial mitochondrial Ca^2+^ uptake rate, correlating with impaired MCU function (57). Transcriptional downregulation: Boudina et al. demonstrated that decreased PGC-1α expression in diabetic myocardium leads to reduced transcription of mitochondrial calcium transporters (including MCU and MICU1) (59). Oxidative damage: Ye, G. et al. proved that persistent ROS generation oxidatively modifies MCU channel subunits, diminishing their activity (30).

Increased release of mitochondrial Ca^2+^ and heightened sensitivity of mPTP are observed.

Experimental evidence demonstrates that in db/db mouse cardiac mitochondria, significantly less Ca^2+^ load is required to induce mPTP opening. Calcium retention capacity (CRC) is decreased. Inhibition of the mNCX improves calcium homeostasis (60). In isolated mitochondria: Mitochondria incubated under high glucose conditions exhibit increased susceptibility to mPTP opening (61). Mechanisms underlying elevated mPTP sensitivity: Upregulation of mNCX: Hyperglycemia activates protein kinase C (PKC) and Rho-associated kinase (ROCK), leading to increased expression and activity of mNCX (62). ROS/RNS-mediated modification of CypD: Accumulation of ROS (particularly H_2_O_2_ generated from superoxide dismutation) and lipid peroxidation products (e.g., 4-HNE) directly modify CypD, significantly lowering the threshold for mPTP opening (61). Increased intracellular [Na^+^]: Inhibition of Na^+^/K^+^-ATPase by advanced glycation end products (AGEs) in late stages elevates intracellular [Na^+^]. This promotes mNCX-mediated Ca^2+^ efflux from mitochondria (63).

##### 3.1.1.1 Decoupling of cytosolic-mitochondrial calcium transients

Reported findings: Confocal imaging (64) (STZ rat cardiomyocytes): Cytosolic Ca^2+^ transients exhibit reduced amplitude and delayed recovery. Mitochondrial Ca^2+^ responses display a more pronounced lag relative to cytosolic signals. Electron microscopy (65) (human diabetic myocardium): Reduced sarcoplasmic reticulum (SR)-mitochondria contact sites are observed. Mechanisms underlying this decoupling include Sarcoplasmic Reticulum (SR) Dysfunction: Hyperglycemia suppresses SERCA2a (66) activity via oxidative modifications and O-GlcNAcylation modifications. Hyperglycemia increases RyR leak due to pathological hyperphosphorylation by PKA/PKC (67). Structural Disruption: Decreased Junctophilin-2 (JPH2) expression in diabetic myocardium increases the coupling distance between SR and mitochondria and impairs the efficiency of microdomain Ca^2+^ transfer (68).

#### 3.1.2 Pathological consequences of mitochondrial Ca^2+^ overload or impaired uptake

The dysregulation of mitochondrial Ca^2+^ homeostasis, whether it manifests as insufficiency, local overload, or signaling decoupling, sets in motion a cascade of pathological events that drive the progression of DCM. As summarized in **Table 1**, these consequences are multifaceted and interlinked.

**Table 1:** Presents the pathological consequences of mitochondrial Ca^2+^ overload or impaired uptake.

Disorder type	Trigger conditions	Core consequences	Disease phenotypes	References
Insufficient Ca^2+^	Persistent hyperglycemia /Insulin resistance	Energy Crisis:↓TCA cycle enzyme activity →↓ATP synthesis →↓Contractility Metabolic Stagnation:↑Fatty acid oxidation + ↓Glucose utilization →Lipotoxic Accumulation	Early Diastolic Dysfunction →Systolic Dysfunction Deterioration	(57–59, 69–71)
Local Ca^2+^ Overload	Stress(e.g.,Ischemia,Sympathetic Excitation)	Oxidative Stress Burst:↑mPTP Opening + ↑ROS→Membrane Lipid/Protein Damage Cell Death: Apoptosis(Cyt c Release) & Necrosis(ATP Depletion)	Myocardial Cell Loss→Fibrosis→Cardiac Chamber Enlargement	(21, 22, 51, 60, 61, 72–75)
Ca^2+^ Signal Decoupling	SR Dysfunction + Structural Damage	Electromechanical Desynchrony: Delayed Cytosolic Ca^2+^ Clearance →Diastolic Dysfunction Worsening Energy Supply Lag: Mitochondria Fail to Respond to Metabolic Demands	Arrhythmia Risk↑ + Exercise Tolerance ↓	(64–68, 76–78)
Inflammatory Activation	mtDNA Release (via mPTP) + ROS Burst + Metabolic Danger Signals	• Inflammasome Activation (e.g., NLRP3) • Pro-inflammatory Cytokine Release (e.g., IL-1β, IL-18) • Immune Cell Infiltration	Myocardial Inflammation → Amplification of Injury & Fibrosis	(40, 73, 75)
Impaired Mitophagy	Loss of Ca^2+^-mediated Activation Signals + Oxidative Damage to Mitophagy Proteins	• Accumulation of Damaged Mitochondria • Aggravated ROS Production & Energy Deficit • Failed Quality Control	Accelerated Cardiomyocyte Senescence & Death	(20, 79)
Mitochondrial Dynamics Imbalance	Ca^2+^-dependent Activation of Fission Proteins (e.g., Drp1) + Reduced Fusion	• Excessive Mitochondrial Fission • Fragmented Mitochondrial Network • Compromised Metabolic Flexibility	Energetic Inefficiency & Increased Susceptibility to Apoptosis	(20, 80)
Sustained ER Stress	Disrupted ER-Mitochondria Ca^2+^ Crosstalk + Unfolded Protein Load	• Activation of UPR Pathways (PERK, IRE1α, ATF6) • Exacerbated Insulin Resistance & Apoptosis • Worsening of Ca^2+^ Handling Defects	Aggravated Contractile Dysfunction & Cell Loss	(79)
Microvascular Dysfunction	Endothelial Cell Mitochondrial Ca^2+^ Dysregulation + AGE/ROS insult	• Reduced NO Bioavailability • Increased Endothelial Permeability & Inflammation • Impaired Vasodilation	Myocardial Ischemia & Perfusion-Contraction Mismatch	(63, 73)

*Ca^2+^, Calcium; ATP, Adenosine Triphosphate; ROS, Reactive Oxygen Species; Cyt c, Cytochrome c; SR, Sarcoplasmic Reticulum; mtDNA, Mitochondrial DNA; IL, Interleukin; mPTP, Mitochondrial Permeability Transition Pore; UPR, Unfolded Protein Response; AGEs, Advanced Glycation End Products; NO, Nitric Oxide.

Insufficient mitochondrial Ca^2+^ uptake, driven by persistent hyperglycemia and insulin resistance, creates a profound energy crisis. The lack of Ca^2+^-mediated activation of TCA cycle dehydrogenases leads to reduced NADH/FADH_2_ production and, consequently, impaired ATP synthesis (69, 70). This bioenergetic deficit directly compromises cardiac contractility (71). Furthermore, this metabolic stagnation promotes a reliance on fatty acid oxidation while suppressing glucose utilization, exacerbating lipotoxic accumulation within the myocardium. Clinically, this manifests initially as early diastolic dysfunction, which progressively deteriorates into overt systolic failure.

Local mitochondrial Ca^2+^ overload, often triggered by acute stressors like ischemia or sympathetic excitation in the diabetic heart, initiates a destructive cycle. The overload induces a massive burst of ROS and sensitizes the mPTP, leading to irreversible opening. This results in oxidative damage to lipids and proteins and the release of pro-apoptotic factors like cytochrome c, triggering both apoptotic and necrotic cell death (74, 75). The ensuing loss of cardiomyocytes is replaced by fibrotic tissue, leading to cardiac chamber enlargement and progressive remodeling.

##### 3.1.2.1 Ca^2+^ Signal decoupling between cytosolic and mitochondrial compartments

DCM severely compromises the efficient transfer of calcium from the sarcoplasmic reticulum (SR) to the mitochondria, which is crucial for matching energy production to demand. This decoupling of Ca^2+^ signals arises from a combination of SR dysfunction and structural damage. Hyperglycemia suppresses SERCA2a activity through oxidative and O-GlcNAcylation modifications, impairing SR Ca^2+^ reuptake and delaying cytosolic Ca^2+^ clearance (66). Concurrently, it promotes pathological hyperphosphorylation of the ryanodine receptor (RyR) by PKA/PKC, increasing its leakiness (67). The downregulation of Junctophilin-2 (JPH2) in the diabetic myocardium increases the physical distance between the SR and mitochondria (68). This combination of functional and structural defects results in electromechanical desynchrony, manifesting as delayed cytosolic Ca^2+^ transients and worsened diastolic dysfunction. Additionally, mitochondria do not detect or respond to cytosolic Ca^2+^ signals, resulting in an energy supply lag that cannot accommodate abrupt metabolic demands, thereby diminishing exercise tolerance and elevating the risk of arrhythmias.

##### 3.1.2.2 Inflammatory activation

Mitochondrial Ca^2+^ dysregulation is a potent trigger of sterile inflammation in DCM. Pathological mPTP opening and ROS bursts can cause the release of mitochondrial DNA (mtDNA) and other damage-associated molecular patterns (DAMPs) into the cytosol. These molecules serve as strong danger signals that activate the inflammasome, especially the NLRP3 inflammasome. This, in turn, catalyzes the maturation and release of potent pro-inflammatory cytokines such as IL-1β and IL-18 and promotes immune cell infiltration into the myocardium. This chronic, low-grade inflammatory activation amplifies initial injury, promotes fibrotic remodeling, and creates a vicious cycle that further deteriorates cardiac function.

##### 3.1.2.3 Impaired mitophagy

The selective removal of damaged mitochondria, known as mitophagy, is essential for maintaining a healthy mitochondrial network. This process is often Ca^2+^-dependent. In DCM, the loss of proper Ca^2+^-mediated activation signals, combined with oxidative damage to key mitophagy proteins (e.g., PINK1/Parkin), impairs mitophagic flux. The resulting accumulation of damaged mitochondria becomes a persistent source of ROS and contributes to the energy deficit. This failure in mitochondrial quality control accelerates cardiomyocyte senescence and death, as the cell is unable to clear its dysfunctional power plants.

##### 3.1.2.4 Mitochondrial dynamics imbalance

Mitochondria exist in a dynamic equilibrium of fission and fusion. Ca^2+^ is a key regulator of this process. In DCM, pathological matrix Ca^2+^ levels promote the activation of fission proteins like Drp1, while fusion processes (mediated by proteins like Mitofusin 2) are often suppressed. This condition leads to a mitochondrial dynamics imbalance skewed towards excessive fission. The result is a fragmented mitochondrial network characterized by small, punctate organelles that are bioenergetically inefficient and more susceptible to apoptosis. This loss of metabolic flexibility and interconnectivity further deepens the energetic crisis in the failing diabetic heart.

##### 3.1.2.5 Sustained ER stress

The endoplasmic reticulum (ER) and mitochondria are physically and functionally connected at mitochondria-associated membranes (MAMs), where they exchange Ca^2+^. Disruption of this ER-mitochondria Ca^2+^ crosstalk in DCM, often due to the structural and functional defects mentioned earlier, leads to sustained ER stress. The accumulation of unfolded proteins in the ER activates the unfolded protein response (UPR) pathways (PERK, IRE1α, ATF6). Chronic UPR activation exacerbates insulin resistance, promotes apoptosis, and further worsens cellular Ca^2+^ handling defects, creating a feed-forward loop of cellular injury and aggravated contractile dysfunction.

##### 3.1.2.6 Microvascular dysfunction

The impact of mitochondrial Ca^2+^ dysregulation extends beyond cardiomyocytes to cardiac endothelial cells. In these cells, similar dysregulation, compounded by insults from AGEs and ROS, contributes to microvascular dysfunction. This manifests as reduced nitric oxide (NO) bioavailability, increased endothelial permeability, inflammation, and impaired vasodilation. The resulting compromise in coronary microcirculation leads to myocardial ischemia and a perfusion-contraction mismatch, further starving cardiomyocytes of oxygen and nutrients and exacerbating the core pathology of DCM.

### 3.2 Key pathways/mechanisms underlying mitochondrial calcium dysregulation in DCM

#### 3.2.1 Energy metabolism impairment

McCormack, J.G., and colleagues demonstrated the reduction in activity of the Ca^2+^-dependent dehydrogenase OGDH (69). Inhibited mitochondrial Ca^2+^ uptake obstructs the TCA cycle (70). This leads to decreased ATP production and consequent reduction in myocardial contractility (71).

##### 3.2.1.1 Exacerbated oxidative stress

**Figure 1:**
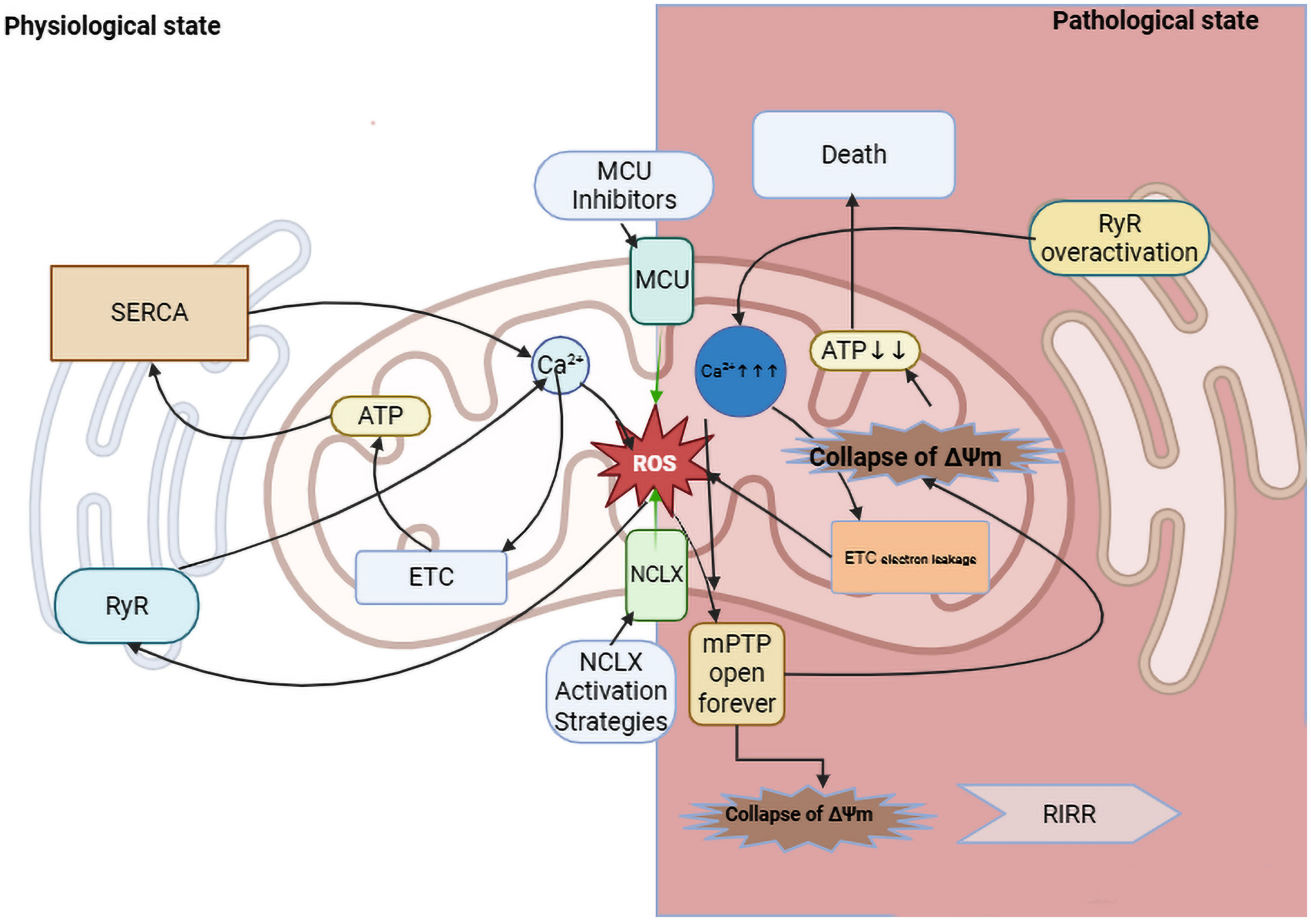
Mechanisms of oxidative stress exacerbation by mitochondrial Ca^2+^ dysregulation in DCM. Ca^2+^, ROS, and ATP engage in a close, dynamic, and bidirectional interplay at both mitochondrial and cellular levels, forming a core pathological network in DCM. Under diabetic conditions, they form a vicious cycle, particularly characterized by Ca^2+^ overload triggering ROS production, which further disrupts Ca^2+^ homeostasis and energy metabolism. Mitochondria serve as the primary site of convergence for these factors. Their internal dynamics—such as mPTP opening and changes in ΔΨm—combined with the unique ROS-induced ROS release (RIRR) mechanism, enable rapid amplification of minor perturbations, ultimately leading to a drastic switch in cellular fate. Potential therapeutic strategies to break this cycle are highlighted: (1) Inhibition of the Mitochondrial Calcium Uniporter (MCU) to reduce pathological Ca^2+^ influx; (2) Activation of the mitochondrial Na^+^/Ca^2+^/Li^+^ Exchanger (NCLX) to enhance Ca^2+^ efflux. MCU, Mitochondrial Calcium Uniporter; NCLX, Mitochondrial Na^+^/Ca^2+^/Li^+^ Exchanger; mPTP, Mitochondrial Permeability Transition Pore; ETC, Electron Transport Chain; SERCA, Sarco/Endoplasmic Reticulum Ca2+-ATPase; ROS, Reactive Oxygen Species; RIRR, ROS-Induced ROSRelease.

Ca^2+^ overload induces excessive ROS generation (72), causing oxidative damage to mitochondrial proteins, lipids, and DNA (73). This establishes a vicious cycle of mitochondrial dysfunction and progressive oxidative injury (81). Mitochondrial calcium dysregulation exacerbates oxidative stress in diabetic cardiomyopathy through converging mechanisms, with the core pathways summarized in **Figure 1**.

##### 3.2.1.2 Pathological mPTP opening

**Figure 2:**
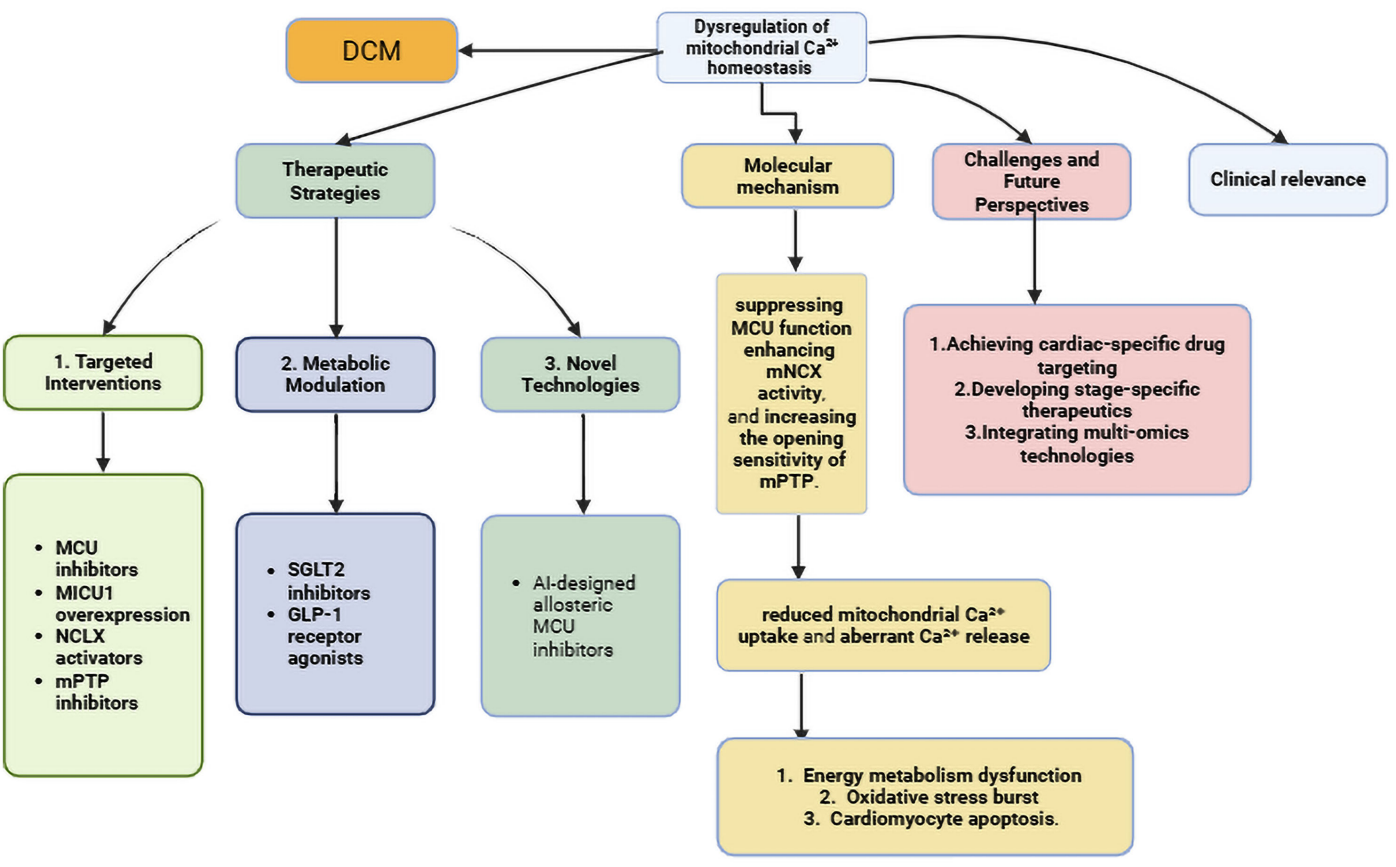
Pathological consequences of mPTP opening in DCM. Ca^2+^ directly binds to CypD and induces its conformational change; the Ca^2+^-CypD complex targets the F-ATP synthase (involving the F-ATP synthase pore-forming hypothesis), leading to mPTP opening and triggering a pathological cascade reaction. Ca^2+^, Calcium; Cyp D, Cyclophilin D; Mptp, Mitochondrial Permeability Transition Pore; ROS, Reactive Oxygen Species.

Ca^2+^ overload serves as the primary trigger for mPTP opening (74). Sustained opening results in mitochondrial swelling and rupture, cytochrome c release, and activation of cardiomyocyte apoptosis and necrosis (75). And myocyte loss and cardiac fibrosis. Pathological opening of the mPTP triggers a lethal cascade in myocardial cells, with the resultant pathological outcomes depicted in **Figure 2**.

##### 3.2.1.3 Abnormal calcium signaling

The interplay between cytosolic and mitochondrial Ca^2+^ handling is crucial. Dysregulation of SERCA2a, as occurs in DCM, delays cytosolic Ca^2+^ clearance during diastole, contributing to diastolic dysfunction. This persistent elevation in cytosolic Ca^2+^ can, in turn, lead to passive, pathological loading of mitochondria, especially if the MCU complex is dysregulated. Recent studies higdemonstrate that in DCM, the coexistence of reduced MCU expression and impaired SERCA2a function leads to a mishandling of Ca both compartments, severely compromising excitation-contraction coupling and overall cardiac performance (76–78, 82).

### 3.3 Pathological interconnections with diabetic metabolic dysregulation

Scholars summarize four major injury axes in diabetes: glucotoxicity, lipotoxicity, oxidative stress, and insulin resistance. Through crosstalk, they collectively suppress the calcium homeostasis network (83).

In greater detail:

Glucotoxicity: Suarez and his team discovered that high glucose activates PKCβ, which phosphorylates the MCU subunit (MiCu1) at Ser^57^, inhibiting calcium uptake capacity (↓40%), leading to cardiomyocyte energy metabolism disorders (84).

Advanced Glycation End Products (AGEs): Bidasee, K. R., and his team found that AGEs, via RAGE activation, inhibit SERCA activity, resulting in passive mitochondrial Ca^2+^ overload (85).

Oxidative Stress: In the pathogenic mechanisms of diabetes, oxidative stress has a significant influence. Seifer, D. R., and his team discovered that the PERK-ATF4 axis downregulates MICU1 expression, causing dysregulated mitochondrial calcium uptake (79). High glucose-induced ROS activates PKCδ to phosphorylate CypD at Ser^191^, lowering the mPTP opening threshold (ΔΨm collapse accelerated 3-fold) (86).

Lipotoxicity and Insulin Resistance: Anderson, E. J., and their team discovered that abnormal fatty acid metabolism inhibits NCLX activity (87).

Collectively, these diabetic injury axes converge on the molecular machinery of mitochondrial Ca^2+^ homeostasis, making it a final common pathway translating systemic metabolic insults into overt cardiac disease.

### 3.4 Clinical translation: diagnostic and therapeutic implications

The dysregulation of mitochondrial Ca^2+^ homeostasis identified in DCM presents significant opportunities for advancing clinical management. Critically, specific dysregulation patterns—such as reduced MCU expression in myocardial biopsies from T2DM patients (58) and heightened mPTP sensitivity in preclinical models (60, 61)—may serve as novel stratification biomarkers. These could identify high-risk individuals progressing from subclinical cardiac dysfunction to overt heart failure, a transition observed in ~29% of asymptomatic T2DM patients within 5 years (7).

However, current clinical tools (e.g., echocardiography, serum BNP) lack the sensitivity to evaluate mitochondrial Ca^2+^ handling directly. Emerging noninvasive techniques—such as hyperpolarized ¹³C-MRI probing calcium-associated metabolic disturbances (88) or mitochondria-targeted PET tracers—hold promise for bridging this gap. It is very important to test these methods against histological gold standards, such as MCU protein quantification in biopsies. Building upon these emerging imaging techniques, a multi-modal biomarker approach is crucial for the early detection, risk stratification, and therapeutic monitoring of DCM. **Table 2** summarizes a range of possible biomarkers based on mitochondrial calcium dysregulation. It compares their detection methods, benefits, and how ready they are for use in clinical settings. The “Translational Readiness Level” (TRL) is scored on a 1–9 scale, where 1–3 indicates basic research, 4–6 indicates clinical assay development/validation, and 7–9 indicates routine clinical application.

**Table 2:** Potential diagnostic biomarkers of mitochondrial calcium dysregulation in diabetic cardiomyopathy.

Category	Target/Marker	Detection method	Key advantages	Translational readiness level (TRL)	Challenges
Tissue	MCU ↓	Biopsy + WB/IHC	Direct pathological readout.	3-4 (Requires invasive procedure)	Invasive
	mPTP sensitivity ↑	Isolated mitochondria assay	Functional, prognostic	3 (Ex vivo validation in human tissue)	Fresh tissue needed.
	NCLX ↓	Biopsy + flux assay	Efflux pathway target	2-3 (Preclinical evidence)	Not standardized
Imaging	TCA-linked metabolism	Hyperpolarized ¹³C-MRI	Non-invasive, real-time	5-6 (Early-phase clinical trials)	High cost, complex
	ΔΨm	PET tracers	Global mitochondrial health	4-5 (Tracer development & preclinical validation)	Limited human validation
	SR-Mitochondria coupling	Super-resolution microscopy	Structural defect detection	2 (Preclinical technology)	Research-only
Blood	ccf-mtDNA	qPCR/ddPCR	Liquid biopsy, serial use	5 (Assay established; clinical correlation ongoing)	Low disease specificity (89, 90)
	MICU1/MCU (plasma)	ELISA (hypothetical)	Minimally invasive	1-2 (Hypothetical/early research)	Unclear tissue correlation

*TRL, Translational Readiness Level; WB, Western Blot; IHC, Immunohistochemistry; CRC, Calcium Retention Capacity; ccf-mtDNA, Circulating Cell-free Mitochondrial DNA; q/ddPCR, Quantitative/Digital Droplet PCR; NGS, Next-Generation Sequencing; ELISA, Enzyme-Linked ImmunosorbentAssay.

Therapeutically, the identification of such biomarkers would enable stage-specific interventions. For instance, patients with predominant MCU downregulation and bioenergetic deficit (e.g., early-stage DCM) might benefit from strategies to augment mitochondrial Ca^2+^uptake. In contrast, those with evidence of heightened mPTP sensitivity or impaired NCLX function (e.g., advanced DCM) could be prioritized for trials with mPTP inhibitors or NCLX activators, respectively. The development of these biomarkers is therefore not merely diagnostic but foundational to a precision medicine approach for DCM.

Therapeutically, stage-specific interventions are warranted:

Early-stage DCM (compensatory phase): Augmenting mitochondrial Ca^2+^uptake [e.g., via AMP-activated Protein Kinase AMPKγ1/PGC-1α axis activation (91)] may correct bioenergetic insufficiency.

Advanced DCM (decompensated phase): Inhibiting mPTP opening (e.g., CypD-targeted agents) or enhancing Ca^2+^ efflux [e.g., NCLX activators (92)] could attenuate cardiomyocyte loss.

Clinical trials should prioritize agents with cardioselectivity [e.g., MCU-i4 (93)] to minimize off-target effects.

## 4 Therapeutic strategies targeting mitochondrial calcium homeostasis

**Table 3** summarizes recent advances in the pharmacological modulation of mitochondrial Ca^2+^ flux. Key strategies include direct MCU inhibition, MICU1 modulation, NCLX activation, mPTP inhibition, targeting, and mitochondrial antioxidants with distinct efficacy and safety profiles.

**Table 3:** Pharmacological strategies targeting mitochondrial calcium homeostasis in cardiac and non-cardiac models.

Target/Compound	Potency (μM)	Model ♥/♦	Key outcome & safety*	Ref.
MCU inhibitor				
Ru265	0.028	♥ MI	Mito-[Ca^2+^] ↓62%; exercise ↓40%	(94)
MCU-i4	0.5	♥ I/R	Infarct ↓35%; no hepato-renal tox.	(93)
MICU1-OE (AAV9)	—	♥ DCM	ATP ↑50%; >2× OE depresses contract.	(95)
NCLX activator				
AAV9-NCLX	—	♥ HF	LVEF ↑25%; efflux ↑70%	(96)
NCLX-273	0.15	♦ kidney	Renal protection; cardiac data NA	(97)
mPTP inhibitor				
TRO40303	—	♥ STEMI	Infarct ↓18%; nephrotoxicity ↑2.4×	(98)
Sanglifehrin A	—	♥ PO	Fibrosis ↓40%; F <5% oral	(99)
Mitochondrial antioxidant				
MitoTEMPO	—	♥ DCM	ROS ↓65%; E/e′ ↓28%	(100)
MitoQ	—	♦ ageing	Mitophagy ↓40% → cardiac damage	(101)

*♥ cardiac; ♦ non-cardiac.

MCU, Mitochondrial Calcium Uniporter; MI, Myocardial Infarction; Ca^2+^, Calcium; I/R, Ischemia/Reperfusion; MICU1, Mitochondrial Calcium Uptake 1; AAV9, Adeno-Associated Virus serotype 9; NCLX, Na+/Ca2+/Li+ Exchanger (Mitochondrial); HF, Heart Failure; LVEF, Left Ventricular Ejection Fraction; mPTP, Mitochondrial Permeability Transition Pore; CsA, Cyclosporine A; STEMI, ST-Elevation Myocardial Infarction; ROS, Reactive Oxygen Species; E/e′, Ratio of early mitral inflow velocity to early diastolic mitral annular velocity; PINK1, PTEN Induced Kinase 1; Parkin, Parkin RBR E3 Ubiquitin Protein Ligase; MitoTEMPO, a mitochondria-targeted antioxidant specifically designed to reduce oxidative stress within mitochondria.

### 4.1 MitoQ: mitochondria-targeted antioxidant

#### 4.1.1 Inhibiting mitochondrial Ca^2+^ overload/restoring physiological uptake

##### 4.1.1.1 MCU inhibitors

Research on MCU inhibitors has yielded progress while also revealing temporary limitations. Xie, Y., and colleagues discovered that the novel inhibitor Ru265 (IC_50_=28 nM) mitigated calcium overload post-myocardial infarction (mitochondrial [Ca^2+^] ↓62%), but >5 mg/kg dosing caused a 40% decline in murine exercise endurance (94). Although not tested in a pure DCM model, the cardiac-specific MCU inhibitor MCU-i4 shows promise in ischemia-reperfusion injury models, a common comorbidity in patients with diabetes. It reduced infarct size by 35% without detectable hepatorenal toxicity (93). Researchers are still looking into the translational potential of MCU inhibition. Recent studies have further elucidated the role of MCU in cardiac physiology and disease, confirming its inhibition as a potent strategy to protect against calcium overload injury in various models, including ischemia-reperfusion (102). A particularly promising development is the emergence of novel, tissue-specific MCU inhibitors designed to minimize systemic side effects, as highlighted in recent preclinical assessments (103). However, the critical challenge remains in achieving a therapeutic window that mitigates pathology without compromising physiological energy production, especially in the chronic setting of DCM.

##### 4.1.1.2 MICU 1/2 functional regulation

Researchers targeting MICU1/2 functional regulation have achieved significant progress. MICU1 overexpression improved mitochondrial function in diabetic cardiomyopathy (ATP ↑50%) by stabilizing cristae structure—an effect distinct from MCU regulation (95). Conversely, Tomar, D., and team found that whole-body MICU1 knockout exacerbated sepsis-induced cardiac dysfunction, while cardiac-specific overexpression required precise dose control (*>2-fold expression induced contractile suppression*) (92).

##### 4.1.1.3 NCLX activation strategies

Research on NCLX activation strategies has become a prominent focus. Luongo, T. S., and colleagues showed that AAV9-NCLX therapy increased the rate at which calcium leaves the mitochondria by 70% and the left ventricular ejection fraction (LVEF) by 25% (P<0.01) in heart failure models (96). Concurrently, studies revealed that the compound NCLX-273 (EC_50_=0.15 μM) selectively activates renal tubular NCLX, mitigating acute kidney injury while exhibiting significantly reduced cardiotoxicity compared to CGP-37157 (97).

##### 4.1.1.3 Inhibition of mPTP opening

Research has revealed the therapeutic potential of mPTP inhibitors, though limitations persist. Atar, D., and team demonstrated that the CsA analog TRO40303 reduced infarct size by 18% (*P=0.03*) in diabetic STEMI patients yet failed to improve long-term LVEF and resulted in a 2.4-fold increase in nephrotoxicity incidence (98). Complementarily, Karch, J.’s group found Sanglifehrin A attenuated myocardial fibrosis by 40%, but its <5% oral bioavailability necessitates nano-carrier delivery systems (99).

##### 4.1.1.4 Mitochondrial-targeted antioxidant therapy

Researchers targeting mitochondrial antioxidants have mitigated Ca^2+^-induced ROS damage with nuanced outcomes. Dare, A. J., and team demonstrated that MitoTEMPO (1.5 mg/kg) reduced mitochondrial ROS by 65% and improved diastolic function (E/e’ ↓28%) in diabetic hearts, yet failed to reverse fibrosis (100). Zielonka, J.’s group uncovered a paradoxical effect: chronic MitoQ administration suppressed mitophagy (PINK1/Parkin pathway ↓40%), accelerating myocardial damage in aging models (101).

##### 4.1.1.5 Counteracting mitochondrial metabolic dysregulation

Current hypoglycemic/lipid-modulating drugs demonstrate potential indirect protective effects on mitochondrial Ca^2+^ homeostasis. Lopaschuk, G. D.’s team demonstrated that empagliflozin reduces mitochondrial calcium overload risk by elevating ketone body β-HB (calcium transient amplitude ↓25%) (104). Pugazhenthi, S.’s group indicated that liraglutide activates PKA, thereby enhancing MCU closure threshold (*calcium overload threshold ↑3-fold*) (105). Foretz, M., and colleagues found that metformin inhibits the MCU complex via AMPK (calcium uptake ↓30%), while potentially exacerbating energy crises in heart failure patients (106).

### 4.2 Emerging next-generation therapeutic strategies

Zaha, V. G., and their team discovered that AAV9-mediated AMPKγ1 overexpression enhances PGC-1α activity, improving calcium handling in DCM (calcium transient decay accelerated by 40%) (91). Rocha, A. G., and their team discovered that delivering a Mitofusin 2 (MFN2) agonist using the mitochondria-targeted peptide SS-31 restores mitochondrial fusion in diabetic cardiomyopathy (fission/fusion ratio decreased by 60%), resulting in a doubled mPTP opening threshold (80).

Comparative analysis of mitochondrial calcium-targeting strategies reveals distinct advantages and limitations. MCU inhibition (e.g., MCU-i4) effectively prevents calcium overload but risks impairing physiological energy production, creating a therapeutic window challenge. In contrast, NCLX activation enhances calcium efflux without compromising uptake, offering a more physiological approach, but it faces specificity hurdles. The translation of these strategies from preclinical models to clinical practice faces several barriers: (1) species differences in calcium handling proteins, (2) the dynamic nature of the diabetic metabolic milieu that alters drug responses, and (3) the heterogeneity of DCM progression stages requiring personalized approaches. Notably, while MCU inhibitors show promise in acute injury models, NCLX activators may be better suited for chronic DCM management where calcium efflux capacity is progressively impaired.

#### 4.2.1 Comparative analysis of mitochondrial calcium-targeting therapeutic strategies

While the array of therapeutic strategies targeting mitochondrial calcium is expanding, a critical and comparative evaluation reveals distinct advantages, limitations, and potential synergies.

##### 4.2.1.1 MCU inhibition vs. NCLX activation

This represents a fundamental philosophical dichotomy in therapeutic approach. MCU inhibition (e.g., with MCU-i4) is primarily a preventive strategy, aiming to block the entry point of calcium during pathological overload, as seen in ischemia-reperfusion injury. Its main strength is that it works well in emergency situations. However, its major limitation is the “therapeutic window” challenge; since MCU is essential for physiological energy production, excessive or chronic inhibition risks exacerbating the bioenergetic deficit that characterizes DCM. In contrast, NCLX activation is a corrective or facilitative strategy. It does not interfere with physiological uptake but enhances the clearance of excess calcium, thereby breaking the cycle of overload. This makes it theoretically more suitable for chronic conditions like DCM, where efflux mechanisms are progressively impaired. The translational hurdle for NCLX activators lies in achieving tissue and context specificity to avoid systemic disturbances in calcium signaling.

##### 4.2.1.2 Stage-specific considerations

The choice of strategy may be critically dependent on the stage of DCM. In early-stage DCM, characterized by impaired calcium uptake and bioenergetic deficit, strategies to augment physiological uptake (e.g., via AMPK/PGC-1α activation) or stabilize the MCU complex might be beneficial. Conversely, in advanced DCM, where calcium overload and mPTP sensitization dominate, MCU inhibitors, NCLX activators, or mPTP blockers would be more logical choices. This underscores the necessity of precision medicine and reliable biomarkers to stratify patients.

##### 4.2.1.3 Combination therapies

Given the complexity of DCM, multitarget approaches may be required. For instance, a combination of a mild MCU modulator (to prevent severe overload) with an NCLX activator (to promote efflux) and a mitochondrial antioxidant (to reduce ROS-triggered mPTP opening) could offer synergistic benefits, potentially at lower, safer doses of each agent. However, the combination also increases the risk of unforeseen off-target effects and necessitates sophisticated pharmacokinetic studies.

## 5 Challenges and future directions

The translation of mitochondrial calcium homeostasis as a therapeutic target from compelling preclinical data to clinical reality for DCM patients is fraught with challenges. This section delineates the principal barriers and outlines promising avenues for future research.

### 5.1 The specificity and safety hurdle of pharmacological intervention

A primary concern in targeting mitochondrial calcium fluxes is achieving therapeutic efficacy without disrupting physiological function. MCU inhibitors exemplify this “double-edged sword” effect. While they potently prevent pathological calcium overload, their chronic use risks exacerbating the pre-existing bioenergetic deficit that characterizes DCM by blunting the calcium signals essential for stimulating ATP production (107, 108). Furthermore, systemic inhibition of a ubiquitous protein like MCU may incur off-target effects in other high-energy-demand tissues, such as skeletal muscle and neurons. Therefore, the development of cardiac-targeted delivery systems (e.g., using cardiotropic viral vectors or nanoparticle carriers) or conditionally activatable prodrugs (designed to be active only in the pathological microenvironment, e.g., during excessive ROS or Ca^2+^ levels) is a critical future direction (93, 107).

### 5.2 Heterogeneity in mitochondrial calcium dysregulation across different stages of DCM

DCM is a progressive disease, and the nature of mitochondrial calcium dysregulation evolves with its stages. In the early compensatory phase, the primary defect is often impaired mitochondrial Ca^2+^ uptake due to downregulation of the MCU complex and PGC-1α. This condition leads to a bioenergetic deficit but not overt overload. The heart may try to make up for it in other ways. However, as the disease advances to the decompensated phase, chronic metabolic stress and cellular damage predispose the myocardium to pathological Ca^2+^ overload. This process is characterized by increased susceptibility to mPTP opening, exacerbated by oxidative stress and the dysregulation of efflux pathways like NCLX. Wu, S., and their team gave us important information by demonstrating stage-specific regulatory mechanisms, such as FBXL4-mediated degradation of MCU in the decompensated phase, which fundamentally alters the mitochondrial calcium handling phenotype (109, 110). This heterogeneity necessitates stage-specific therapeutic interventions.

### 5.3 Bridging the translational gap: from animal models to human DCM

A significant barrier to clinical progress is the limited predictive value of current preclinical models. Rodent models of diabetes (e.g., db/db mice, STZ rats) capture certain metabolic features but fail to fully recapitulate the chronic, multi-factorial nature of human DCM, which develops over decades amidst complex genetic backgrounds and comorbidities.

Key limitations include:

- Species Differences: The expression, regulation, and relative importance of proteins like MCU and NCLX can differ between rodents and humans.
- Model Uniformity vs. Patient Heterogeneity: Genetically uniform animal models do not reflect the vast heterogeneity of human DCM, leading to over-optimistic drug efficacy that fails in diverse clinical populations.
- The Dynamic Diabetic Milieu: Preclinical testing often occurs in a stable metabolic state, unlike the fluctuating glucose, insulin, and adipokine levels in patients, which can profoundly alter drug responses.
- To bridge this gap, future research must prioritize:
- Human-Relevant Models: Utilizing induced pluripotent stem cell-derived cardiomyocytes (iPSC-CMs) from diabetic patients with and without cardiomyopathy.
- Comorbidity-Incorporated Designs: Testing drug efficacy in models that combine diabetes with other common comorbidities like hypertension or aging.
- Biomarker-Driven Stratification: The discovery and validation of non-invasive biomarkers (as proposed in **Table 2**) are paramount for identifying responsive patient subpopulations in clinical trials.

### 5.4 The rationale for multitarget combination therapies

Given the complex, interconnected pathology of DCM, simultaneously targeting multiple nodes in the mitochondrial calcium network may yield synergistic benefits. Hamilton, S., and colleagues provided a proof-of-concept, demonstrating that dual inhibition of the inositol trisphosphate receptor (IP3R) and MCU was more effective in modulating mitochondrial Ca^2+^ uptake in heart failure than either approach alone (111). Logical combinations for DCM could include a mild MCU modulator (to prevent severe overload) with an NCLX activator (to enhance clearance) and a mitochondrial antioxidant (to reduce ROS-mediated mPTP sensitization). However, such strategies require sophisticated pharmacokinetic and safety studies to avoid unforeseen off-target effects.

### 5.5 Harnessing novel technologies for mechanistic insight and drug discovery

Cutting-edge technologies stand poised to transform the future of DCM research and therapy development:

- Single-Cell and Spatial Omics: Technologies like scRNA-seq are already illuminating the cellular heterogeneity of the failing heart, identifying distinct cardiomyocyte subpopulations with unique calcium handling gene expression profiles (112). This makes it possible to find new cell-specific targets and biomarkers.
- Advanced Imaging: Real-time mitochondrial calcium monitoring with improved genetically encoded indicators (e.g., mtGCaMP) and hyperpolarized ¹³C-MRI, which can probe calcium-related metabolic fluxes, offer non-invasive windows into mitochondrial function *in vivo* (88, 113).
- Artificial Intelligence (AI): AI-driven drug design is breaking new ground in targeting complex proteins, like the MCU complex (114). AI-designed allosteric inhibitors have demonstrated effectiveness in alleviating mitochondrial calcium overload in heart failure models (115), and quantum machine learning is currently facilitating the precise prediction of drug-channel interactions (116).

## 6 Conclusions

Mitochondrial calcium homeostasis has emerged from the periphery to claim its position as a central integrator in the pathogenesis of diabetic cardiomyopathy. It functions as a critical signaling nexus, translating the systemic metabolic insults of diabetes—hyperglycemia, lipotoxicity, oxidative stress, and insulin resistance—into the cardinal features of myocardial dysfunction: bioenergetic deficit, oxidative injury, and cardiomyocyte loss. The diabetic environment directly attacks the molecular machinery that controls mitochondrial calcium. This causes problems with uptake through the MCU complex, sensitized efflux through the mPTP, and dysregulated extrusion through NCLX.

The therapeutic landscape is evolving to target this hub, but our analysis reveals that the choice of strategy is not trivial. The fundamental dichotomy between MCU inhibition (a preventive strategy against overload) and NCLX activation (a corrective strategy to enhance efflux) represents a critical trade-off between preventing toxicity and maintaining physiological energy production. The optimal choice varies depending on the stage of DCM and the predominant nature of the calcium handling defect in a particular patient.

Therefore, the path forward must be guided by precision medicine. Success will depend on our ability to leverage emerging technologies—from single-cell omics and AI to advanced imaging—to develop reliable biomarkers for patient stratification and to design cardioselective therapies that are effective within the complex, dynamic metabolic context of diabetes. Ultimately, safeguarding the mitochondrial calcium gateway may be the key to protecting the diabetic heart and altering the devastating cardiovascular destiny of millions of patients worldwide.
